# Development of a large SNPs resource and a low-density SNP array for brown trout (*Salmo trutta*) population genetics

**DOI:** 10.1186/s12864-019-5958-9

**Published:** 2019-07-15

**Authors:** Keoni Saint-Pé, Maeva Leitwein, Laurence Tissot, Nicolas Poulet, Bruno Guinand, Patrick Berrebi, Geoffrey Marselli, Jean-Marc Lascaux, Pierre-Alexandre Gagnaire, Simon Blanchet

**Affiliations:** 1 Centre National de la Recherche Scientifique (CNRS), Université Paul Sabatier (UPS), Station d’Ecologie Théorique et Expérimentale, SETE, UMR 5321, 2 route du CNRS, 09200 Moulis, France; 2 Département de Biologie, Institut de Biologie Intégrative et des Systèmes (IBIS), Université Laval, Québec, QC Canada; 3 ISEM, CNRS, Univ. Montpellier, IRD, EPHE, Montpellier, France; 4 Present address: Genome - Research and Diagnostic, 697 avenue de Lunel, 34400 Saint-Just, France; 5 EDF R and D LNHE - Laboratoire National d’Hydraulique et Environnement, 6 quai Watier, 78401 Chatou Cedex, France; 6 Pôle écohydraulique AFB-IMT, allée du Pr Camille Soula, 31400 Toulouse, France; 7 ECOGEA, 352 Avenue Roger Tissandié, 31600 Muret, France; 8 Université de Toulouse, UPS, UMR 5174 (EDB), 118 route de Narbonne, F-31062 Toulouse cedex 4, France

**Keywords:** Conservation, Management, Population genomics, Microarray, Admixture

## Abstract

**Background:**

The brown trout (*Salmo trutta*) is an economically and ecologically important species for which population genetic monitoring is frequently performed. The most commonly used genetic markers for this species are microsatellites and mitochondrial markers that lack replicability among laboratories, and a large genome coverage. An alternative that may be particularly efficient and universal is the development of small to large panels of Single Nucleotide Polymorphism markers (SNPs). Here, we used Restriction site Associated DNA sequences (RADs) markers to identify a set of 12,204 informative SNPs positioned on the brown trout linkage map and suitable for population genetics studies. Then, we used this novel resource to develop a cost-effective array of 192 SNPs (96 × 2) evenly spread on this map. This array was tested for genotyping success in five independent rivers occupied by two main brown trout evolutionary lineages (Atlantic -AT- and Mediterranean -ME-) on a total of 1862 individuals. Moreover, inference of admixture rate with domestic strains and population differentiation were assessed for a small river system (the Taurion River, 190 individuals) and results were compared to a panel of 13 microsatellites.

**Results:**

A high genotyping success was observed for all rivers (< 1% of non-genotyped loci per individual), although some initially used SNP failed to be amplified, probably because of mutations in primers, and were replaced. These SNPs permitted to identify patterns of isolation-by-distance for some rivers. Finally, we found that microsatellite and SNP markers yielded very similar patterns for population differentiation and admixture assessments, with SNPs having better ability to detect introgression and differentiation.

**Conclusions:**

The novel resources provided here opens new perspectives for universality and genome-wide studies in brown trout populations.

**Electronic supplementary material:**

The online version of this article (10.1186/s12864-019-5958-9) contains supplementary material, which is available to authorized users.

## Background

The brown trout (*Salmo trutta*) is one of the most widespread freshwater fish species in Eurasia, and it has been widely introduced in both the southern and northern hemispheres [1]. As part of the Salmonidae family, it is a scientifically interesting species because of its diversity in terms of ecology, life history strategies and habitat use [2, 3]. Thanks to its wide ecological variability and excellent ability to spread and colonize new watersheds, the species is found both in fresh and salt waters over most of its range. The brown trout is also an economically major species in terms of farming, net fishing (for the sea-run form), and expenditure in recreational angling [4, 5], partly explaining its worldwide intentional introduction [6]. Because this species is strongly associated to human interests, wild brown trout populations are widely managed, either to sustain attractive leisure activities such as recreational angling or to conserve declining and/or emblematic populations. Moreover, the brown trout has been domesticated since the nineteenth century [7], and hatchery strains have been largely used to sustain wild populations worldwide [8–10]. Instead of positive expected effects of these stocking activities, most have proven to have negative long term effects on wild brown trout populations in part because of the reduced fitness brought by hatchery fish in wild populations, and the loss of local genetic heritage caused by the replacement of local wild populations with genetically homogeneous hatchery strains [11–14].

The brown trout presents high levels of phenotypic and genetic polymorphism, with seven main mitochondrial (mtDNA) lineages with various geographical extents being generally recognized. These consist of (i) four sub-continental lineages: the Atlantic (AT), Mediterranean (ME), Danubian (DA) and Adriatic (AD) lineages, (ii) two regional lineages: Marmoratus (MA) in the north of the Adriatic Sea and North African (NA) in Morocco, Algeria and Sicily [15, 16] and “(iii) two local lineages limited to specific geographical areas: the Duero (DU) in Northwestern Iberian basins, and the Tigris (TI) in Turkey” [17–21]. Within these extent lineages that cover the whole range of *S. trutta*, high levels of genetic and phenotypic polymorphism are also observed at more local spatial scales within lineages (e.g. population scale; [22–24]). However, diversity patterns in brown trout have also been locally influenced by stocking practices that mostly relied on European hatchery strains of AT origin [25] to supplement local populations, with the exception of a few local strains stemming from local populations [26–28].

Genetic tools appeared as a key approach for scientists and local managers to optimize conservation efforts [29, 30] because they provide insight into both the ecological and the evolutionary dynamics of wild populations [10, 31]. For instance, assignment tests, fine-scale population structure, kinship analyses and genome-wide surveys [32–34] enable to monitor populations effectively, and have high potential applications for conservation and management in salmonids, including the brown trout [13, 28, 35].

Molecular studies on trout populations first used allozymes, mitochondrial markers, and then microsatellite loci [7, 36, 37]. These markers (notably microsatellites) are useful and adequate to answer many biological questions, but their genome coverage is generally weak, and replicability and universality are relatively low since each research group generally uses its own panel of markers. Single nucleotide polymorphisms (SNP) markers have been shown to potentially reduce these limitations [38–40]. They allow to uncover a relatively high number of annotated and mapped markers with low scoring-error rates [41, 42]. Also, SNPs markers can easily be chosen to represent both neutral genomic regions and regions under selection, at a genome-wide scale and across large samples [43]. Despite being biallelic markers, SNPs can be highly informative for most analyses used in population genetics, as far as the number of loci is sufficiently high and evenly spread across the genome (> 50; [44–48]). Genome coverage is an important aspect in the choice of markers for population genetics: first, markers evenly spread across the genome are less likely to exhibit linkage disequilibrium [49], and second, it was shown that many population events, such as introgressive hybridization can only concern certain genomic blocks, a full coverage thus enables to capture these events [50, 51].

SNP arrays are commonly used for conservation purposes in salmonids [52–54], although rarely in brown trout, which is probably because only a handful of SNP markers were available for this species [55–57]. However, higher density resources were more recently developed. In particular, Linløkken [58] developed 3781 SNPs to analyse genetic differences between wild and hatchery brown trout in a tributary of Lake Savalen in central Norway. Moreover, a new lineage-specific high density linkage map for *S. trutta* comprising ancestry informative SNPs for both Atlantic (AT) and Mediterranean (ME) evolutionary lineages from Western Europe was also developed [14, 59]. This latter resource provides a novel baseline for the development of mapped SNPs that may be of prime interest for studies on brown trout population genetics across a wide spatial range.

The aim of this study was to develop a genome-wide, mapped and universal set of SNPs for the brown trout for both Atlantic and Mediterranean lineages (AT and ME lineages; sensu Bernatchez, 2001), which would be a useful and affordable tool for both scientists and environmental managers. By taking advantage of the new genomic resource available for brown trout [14, 51, 59], we developed a panel of 12,204 RADs containing 1 or 2 polymorphic SNPs evenly spread across the genome structured in 40 chromosomes, and ancestry informative for at least two of the main brown trout lineages, the Atlantic and the Mediterranean lineages. Among these, a sub-panel of 192 SNPs describing the whole genome was included in a low-density SNP array. The validity of this low-density SNP array was tested by quantifying genotyping success and number of polymorphic loci in five independent populations from the two lineages at a large spatial scale. Finally, the power of this array was compared to a panel of 13 microsatellites for answering classical population genetics questions: admixture with stocked domestic individuals and genetic differentiation among wild populations. All resources are made freely available for future users (Additional file 1: S1 and S2).

## Results

### Development of the large SNPs resource and characteristics of the low-density SNP array

**Fig. 1:**
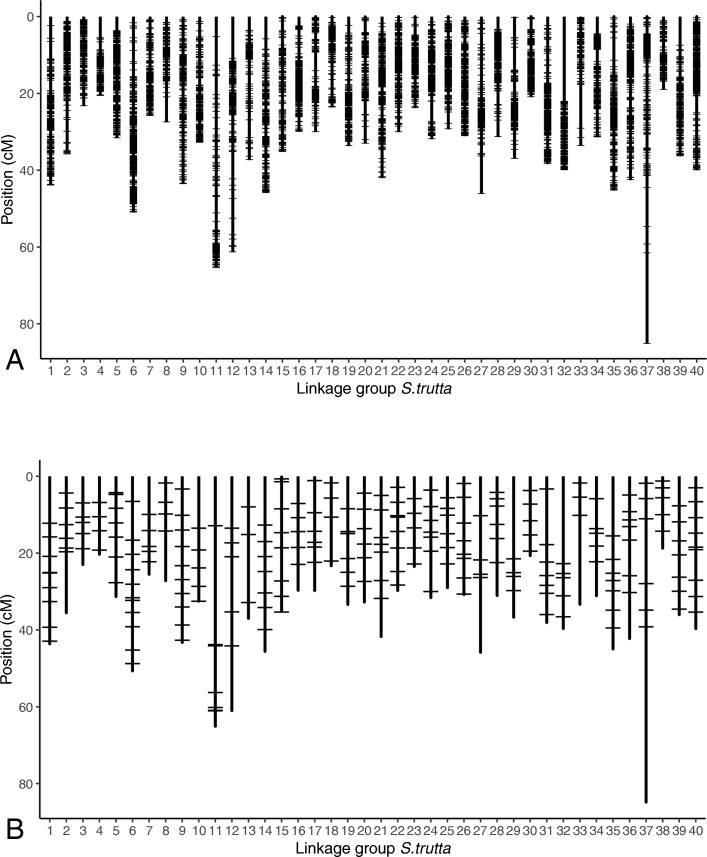
Positions on the brown trout linkage map of the 12204 RADs (**a**; containing one or two SNPs with MAF ≥ 5% based on all individuals (AT + ME) and positions between the first or last 30 bp of the RAD with no undetermined nucleotides), and of the 245 SNPs (**b**; informative for at least the AT and ME lineages, positions between the first or last 50 bp of RADs (primer design zones), and spaced by at least 3.5 cM)

After applying filters, we identified a RAD data set of 12,204 sequences each containing one or two SNPs that met our specifications and that are made readily available to the scientific community (see Additional file 1: S1). The number of RADs per linkage groups (LGs) varies between 137 (LG 33) and 563 (LG 6). These RAD tags are spaced by 0.119 cM (+/− 0.039) in average. They were found in all LGs and they cover the linkage map relatively evenly, although there were large gaps on LGs 11 (top), 12 (top and bottom), 27 (bottom), 32 (top), 33 (bottom), 37 (bottom), and 39 (top) (Fig. 1a). One estimate of the local recombination rate for each SNP (i.e. one index of the relative power of markers for LD-based mapping approaches) is provided in Additional file 1: S1 and S2.

The 245 SNPs selected from this set of RADs were spread over all linkage group so as to cover the linkage map as homogeneously as possible (Fig. 1b). The final low-density 182 SNP array, to which we added 10 SNPs previously developed (see the Methods) is presented in Additional file 1: S2.

### Genotyping success of the low-density SNPs array

Thirty of the 192 SNPs (among which a mitochondrial marker) that were initially genotyped in the five watersheds did not amplify, suggesting primers failure to properly bind their target DNA site. For the loci that successfully amplified, the overall genotyping success was high, with less than 1% of missing data per individual, irrespective of the river basin and the lineage (Table 1). Nonetheless, the number of polymorphic SNPs greatly varied from one river to another, ranging from 91 (for the Seuge River) to 160 (for the Aude River) out of the 162 SNPs that were successfully genotyped in that portion of our work (Table 1). The isolation-by-distance patterns were significant (pairwise Fst significantly correlated to pairwise riparian distance) for the Seuge River (r_Mantel_ = 0.55, *p*-value < 0.001) and the Roya River (r_Mantel_ = 0.65, p-value = 0.01). Fst and riparian distance were also positively although non-significantly correlated for the Aude River (r_Mantel_ = 0.19, p-value = 0.15), the One River (r_Mantel_ = 0.28, p-value = 0.07) and the Doron River (r_Mantel_ = 0.02, p-value = 0.36) (see Additional file 1: S3 for the plots).

**Table 1:** Number of polymorphic loci, number of non-genotyped loci per individual considering polymorphic loci (average, min and max), expected heterozygosity (He) on polymorphic loci in each of the five river basins, and surface drained by the river basin (upstream from the lowest sampling site)

River basin (lineage)	Number of polymorphic loci	Average (±SD) number of non-genotyped loci per individual on polymorphic loci Min-max	He on polymorphic loci	Basin surface (Km^2^)
Aude (Mediteranean)	160/162	1.04 (±1.68) 0–24	0.16	240
Doron de Bozel (Mediteranean)	153/162	0.71 (±0.99) 0–8	0.30	180
One (Atlantic)	119/162	0.87 (±1.23) 0–13	0.14	155
Roya (Mediteranean)	158/162	1.36 (±4.51) 0–63	0.30	360
Seuge (Atlantic)	91/162	0.66 (±1.06) 0–15	0.09	90
Average	136	0.95	0.20	205

### Efficiency of the low-density SNPs array

Among the 30 replaced SNPs for the empirical test in the Taurion River, 10 failed at the amplification step (see Additional file 1: S2). This resulted in a set of 182 markers that successfully amplified, although only 92 of them were polymorphic in this river (among which only one mitochondrial marker of the 10 INRA and mitochondrial added markers). After removing individuals with more than a third of missing data, and keeping only individuals for which we had both SNPs and microsatellite data, our final dataset comprised 197 individuals (167 individuals from the Taurion basin and 30 from the Soueich trout farm). Expected heterozygosity ranged from 0.18 to 0.22 for SNPs and from 0.58 to 0.73 for microsatellites. Fst ranged from 0.022 to 0.070 for SNPs and from 0.019 to 0.040 for microsatellites (details for each site are shown in Table 2). We found one marker that was a significant Fst outlier, suggesting it may be influenced by selection (ID 295415; Fst> > Expected heterozygosity), but we decided to keep it in our analyses (see the Discussion).

**Table 2:** For each site, sample size (“N”), mean expected heterozygosity over all loci (“He”) and standard deviation between loci, mean observed heterozygosity over all loci (“Ho”) and standard deviation between loci, mean allelic richness computed using a rarefaction approach over all loci (“Ar”), mean Fis over all loci (“Fis”), mean Fst over all loci (i.e. uniqueness at the site level; Fst = 1-He_Site_/He_Total_)

		*SNPs*					*Microsatellites*				
Site	N	Ho	He	Fis	Fst	Ar	Ho	He	Fis	Fst	Ar
BEA-Rau	27	0.18 ± 0.19	0.18 ± 0.18	0.011	0.070	1.58	0.44 ± 0.20	0.58 ± 0.25	0.226	0.04	4.42
PON-Rau	12	0.22 ± 0.23	0.21 ± 0.20	−0.038	0.030	1.66	0.67 ± 0.19	0.70 ± 0.17	0.027	0.022	5.63
THA-Bar	26	0.22 ± 0.23	0.22 ± 0.20	−0.025	0.022	1.65	0.65 ± 0.17	0.67 ± 0.18	0.015	0.017	5.85
THA-Tcc	30	0.19 ± 0.20	0.20 ± 0.18	0.028	0.027	1.69	0.61 ± 0.18	0.69 ± 0.17	0.107	0.019	6.18
THA-Usi	29	0.22 ± 0.22	0.21 ± 0.19	−0.023	0.025	1.67	0.68 ± 0.12	0.73 ± 0.16	0.057	0.019	6.79
THA-Vig	21	0.21 ± 0.21	0.22 ± 0.20	0.056	0.028	1.68	0.64 ± 0.17	0.72 ± 0.12	0.125	0.024	6.40
VIG-Tex	22	0.21 ± 0.21	0.21 ± 0.19	−0.004	0.031	1.66	0.69 ± 0.14	0.70 ± 0.15	0.033	0.02	5.91

**Fig. 2:**
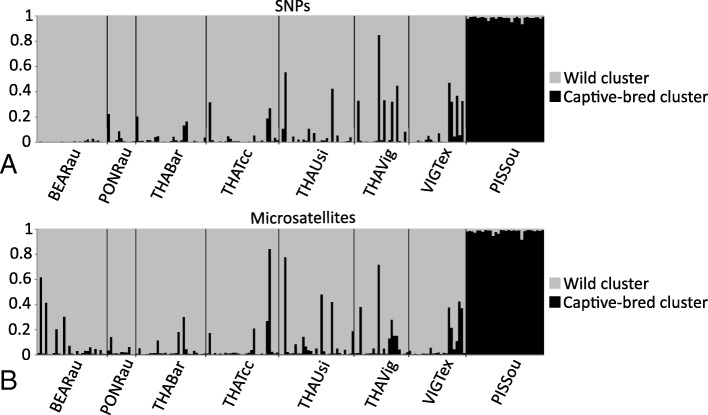
Structure barplots of assignment to the wild (grey) and the captive-bred (black) clusters, using both SNPs (**a**) and microsatellites (**b**)

**Fig. 3:**
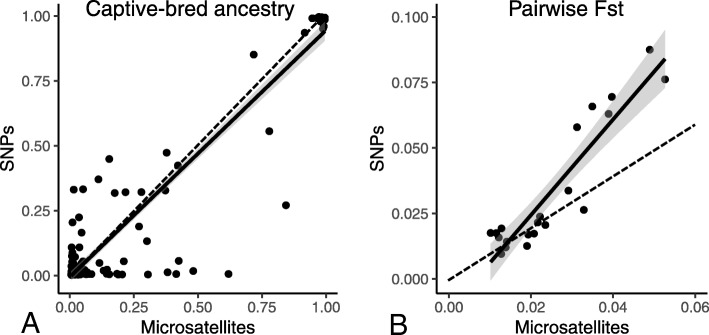
Plot of individual hatchery ancestry proportion measured with microsatellites against that measured with SNPs (**a**) and pairwise Fst between sites measured with microsatellites against pairwise Fst between sites measured with SNPs (**b**). Black lines represent the linear regression with its confidence interval; spotted lines represent the 1:1 line

Individual inferences of hatchery ancestry measured with either SNPs or microsatellites are presented in Fig. 2 in the form of individual barplots. The distribution of captive-bred ancestry was bimodal, meaning that most individuals were either purely wild or captive-bred, with relatively low numbers of admixed genotypes (Fig. 2, Fig. 3a). Levels of hatchery ancestry were significantly and moderately correlated (r_Spearman_ = 0.60, d.f. = 193, *p* < 0.001; Fig. 3a), although for some individuals, there was a discrepancy between markers with one of the two marker types detecting introgressed fish while the other marker type assigned them as pure wild fish (Figs. 2, 3a). Pairwise Fst values are presented for both SNPs and microsatellites in Additional file 1: S4. They ranged from 0.010 to 0.088 for SNPs (mean = 0.033 ± 0.025) and from 0.010 to 0.053 for microsatellites (mean = 0.025 ± 0.013). Pairwise Fst values between sites assessed with SNPs and microsatellites were strongly correlated (r_Mantel_ = 0.92, *p* = 0.001 based on 1000 permutations), and SNPs had higher values (Fig. 3b, the regression coefficient is significantly higher than the 1:1 line (dotted line on figure) since its 95% CI is 1.46–2.18).

**Fig. 4:**
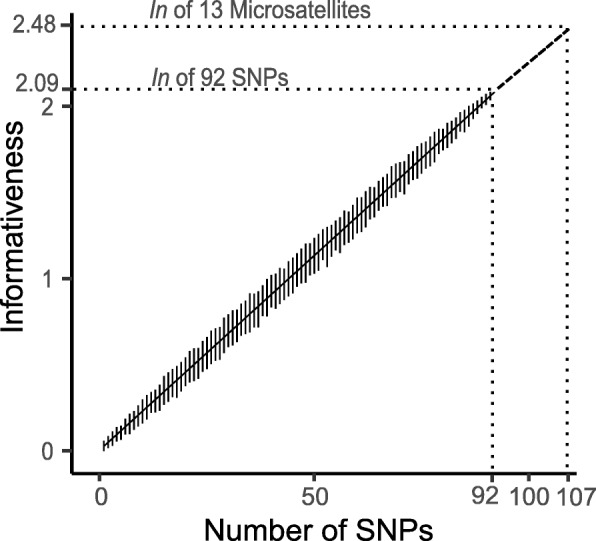
Plot of informativeness against number of SNPs. 107 SNPs would be equivalent to the 13 microsatellites in terms of informativeness

Finally, within the Taurion River, the 92 SNPs displayed a slightly lower informativeness (I) than the 13 microsatellites (2.08 vs. 2.48; Fig. 4). We found that the informativeness of these 92 SNPs is actually equivalent to that of 10 of the microsatellites (Fig. 4). Based on the equation linking the number of our SNPs and informativeness (I = 0.023*Nsnps+ 0.0038; r^2^ = 0.98, p-value < 0.001), we extrapolated that 108 SNPs are required to be equivalent to the panel of 13 microsatellites in terms of informativeness for individual assignment.

## Discussion

The SNP panel developed here was shown to be efficient to study the population genetics of Atlantic and Mediterranean brown trout lineages from Western Europe, which gathered a huge number of studies in the past decades [60–62]. We provided a panel of 12,204 RADs which are relatively evenly spread across the whole genome, and, from a sample of this panel, we proved its efficiency in terms of genotyping success, and measuring patterns of isolation-by-distance. We also proved that these SNPs successfully detect population structure, which opens new insights for many applications with great potential compared to commonly used markers. These advantages are multiple, going from lower error rates and a simple mutation model with low homoplasy [43] to usability on poor quality samples. In terms of costs, SNPs and microsatellites are roughly similar, however in terms of time efficiency, SNPs are highly advantageous: samples are directly sent to the genotyping platform, and within a month, data is ready to be analyzed. Moreover, SNPs can be easily reused in other studies, and are more powerful in detecting hybridization [63].

### Development of a large variation map for population genomic studies in brown trout

The 12,204 RAD-derived SNPs were relatively homogeneously distributed across the 40 linkage groups of *Salmo trutta* (with 137 to 563 RADs per LG), and showed an average spacing of 0.119 cM. There are some gaps in coverage, which does not necessarily mean that information is missing, they could be due to high recombination rates in these regions or to the positions of the centromeres. Although these gaps exist, genome coverage is satisfying, and with the marker density obtained, it should be sufficient for most genome-scale studies that need to tag a large fraction of genomic variation through linkage disequilibrium. However, this marker density may still be limited when a rapid decay of linkage disequilibrium leaves many genomic regions unattainable, as it might be the case for some applications like the search for loci underlying fitness in the wild [64]. Therefore, an estimate of the local recombination rate for each SNP as an index of the relative power of markers for LD-based mapping approaches is provided in Additional file 1: S1 and S2.

The proposed SNP resource only includes SNPs with a MAF higher than 0.05 (i.e. removing of rare variants), a criterion that has been set for two main reasons. First, SNPs with very low MAF can in many cases be genotyping errors [65, 66]. Second, we chose a relatively high MAF because this panel of SNPs is primarily designed for studying populations for most of the species range. Therefore, SNPs that are discovered in the populations used to develop the panel are more likely to be polymorphic in other populations from which they were not developed if their MAF is high [67]. Although filtering SNPs on their MAF could lead to ascertainment bias [40, 68, 69], we suggest this is not an issue in our case study because we applied the MAF criteria regarding distinct glacial lineages, which limits marker choice bias. Moreover, for individually-centered investigations such as population structure, kinship and individual assignment, SNPs with higher MAF were shown to generally be the most powerful [42, 70, 71].

In the test panel, and for the Taurion river (test basin), one locus might be potentially affected by selection. Outlier markers are usually removed before analyses for inferring neutral evolutionary processes, such as genetic drift and gene flow [72]. In our analyses, we decided not to remove it because in the case of detection of admixture between strains, it was shown that these ancestry informative markers can increase accuracy for detecting differentiation and assignment of individuals to populations [68, 73, 74]. Removing loci displaying selection is thus up to the users of the resource, depending on the aim of the study. For instance, if the aim is to determine if a population is at HWE, or to quantify gene flow/inbreeding, or calculate effective population sizes, loci affected by selection should be removed, whereas more individual-based questions do not necessarily require removing these loci.

### Genotyping success

Genotyping success was very high in all basins, and except for two basins, the number of polymorphic SNPs among the 162 amplified (from the 192 set) was satisfactory (73 to 99%), confirming the potential versatility of this tool. This set of 162 SNPs already benefits to the field of population genetics for the species as to our knowledge, most studies used less than 40 SNPs [46–48]. Therefore, the 12,204 RAD panel is a promising tool for genome wide studies on brown trout. It would be of interest however to further test this panel on other evolutionary lineages, or on populations which have been shown to have diverged from the continental populations such as western Mediterranean populations found in Iberian Peninsula [25], Italian populations [75], and Corsican populations [76], as well as on the other main lineages (e.g. Adriatic and Danube lineages; [15]) or remote populations inhabiting at the edges of the species’ range (Iran [77, 78], Morocco [16]).

As a first approach we found this panel to be efficient for detecting patterns of isolation-by-distance, although we had no other markers to compare with. Patterns of isolation-by-distance were found to be significant only in the Seuge and the Roya Rivers, although there was a tendency in the other three basins. Interestingly, it seems that even when the number of polymorphic SNPs was low (Seuge River: 92 polymorphic loci), detecting a pattern of isolation-by-distance was still possible. In other basins, we suggest that the strength of the relation between genetic and riparian distance may also be affected by stocking events, or by characteristics of the watershed and demographic histories. However, we did not investigate these issues further in the present manuscript.

### Tests of the low-density SNP array

**Fig. 5:**
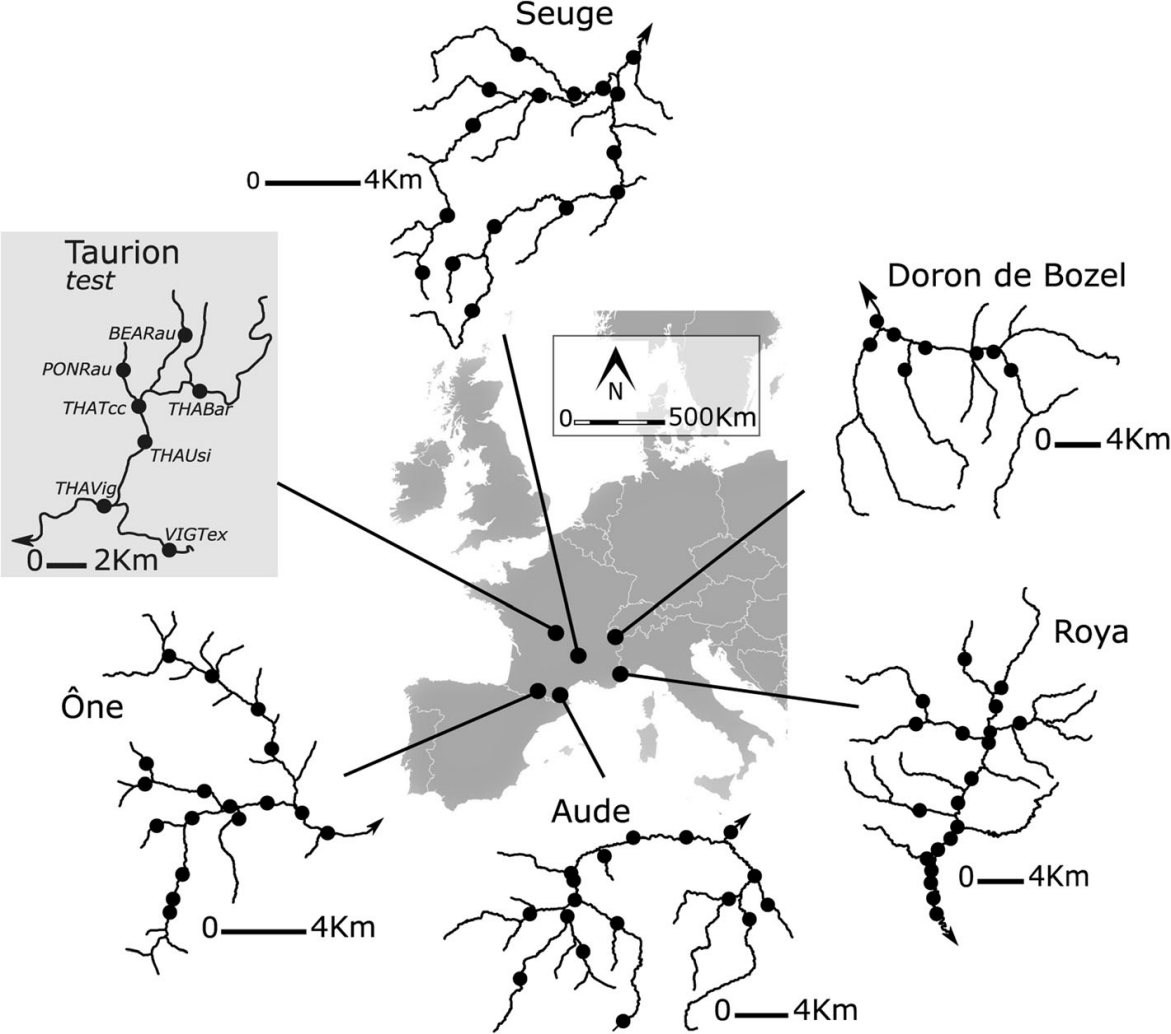
Map of the five river basins and and sampling sites (black dots) used to test for genotyping success. The Seuge and Ône Rivers are part of the Atlantic catchment, therefore naturally harboring AT trout, whereas the Aude, Roya and Doron de Bozel Rivers are part of the Mediterranean catchment, naturally populated with ME lineage. Maps were generated by authors on ArcGis and assembled using Inkscape

Studies in which low-density SNP arrays equal or outperform a handful of microsatellites for population structure and differentiation are common, particularly when sample sizes are large and populations are strongly structured [47, 79, 80]. Although we found no literature on this particular aspect in brown trout, it has been shown in Atlantic Salmon: genetic divergence, structuring and isolation-by-distance were assessed as successfully using only 7 SNPs and 14 microsatellites, although genetic diversity estimates were less concordant [81]. Twenty-six SNPs were also shown to be nearly as efficient as 16 microsatellites for parentage assignment in this species [82], which makes our set of SNPs appear very promising. The panel of 192 SNPs tested here performed well in terms of detection of admixture and population differentiation, although only 92 SNPs were polymorphic in the Taurion basin. The low level of polymorphism is probably due to the fact that the study scale is extremely low (less than 15 km between the two most extreme sampling sites; Fig. 5) and/or that the biogeographic area in which this river basin is situated has historically low level of diversity (see below).

We found similar introgression level when measured with SNPs compared to microsatellites (Fig. 3a), and higher pairwise Fst values between sites with SNPs, suggesting that SNPs have – at least - a similar discriminatory and assignment power. However, the low number of polymorphic SNPs in this panel (92 polymorphic SNPs) compared to other SNP-microsatellite comparison case studies [45, 79, 83] lowered its’ informativeness: it was outperformed by the 13 microsatellites, and we found that it would actually require 107 SNPs to be equivalent to microsatellites in terms of informativeness for assignment. However, the advantage of SNPs may here not be accounted for when calculating informativeness. Indeed, they enable to better detect introgression and admixture compared to microsatellites, and show that individuals considered as « pure » with microsatellites may in fact be introgressed [84]. This difference in individual admixture proportions calculated with microsatellites and SNPs might results from the fact that our panel is characterized by an even repartition of SNPs along the genome of the brown trout, which is expected to improve the global assessment of genome-wide admixture proportions [51]. Hence, the strong advantage of a SNP panel of this type is that it ensures a better representativeness of the entire genome of the brown trout. Moreover, although informativeness is lower, the 92 SNPs still give sufficient information on admixture and differentiation, highly correlated with that given by the microsatellites: the trade-off between cost and power must also be taken into account in regard of the questions asked and the means of the user.

For other empirical case studies, such as the five river basins on which genotyping success was measured, and in which individuals are variable on more loci (except for the Seuge river, other basins showed 119 to 160 polymorphic loci, Table 1), we can expect higher informativeness. We even expect SNPs to equal or outperform microsatellites in terms of informativeness for these basins (indeed, around 107 SNPs should be equivalent to the panel of 13 microsatellites, as pairwise Fst values were higher for SNPs than microsatellites), and with the advantages of SNPs mentioned previously. This is particularly true in Mediterranean rivers, probably because they contain both domestic Atlantic and natural Mediterranean ancestries. Indeed, these SNPs were discovered using a mix of Mediterranean and Atlantic individuals, and are therefore more likely to be polymorphic if both lineages are present. Additionally, the mitochondrial and INRA markers were also developed to have a fixed allele in each lineage, explaining why in the Taurion for instance, in which only the Atlantic lineage is present, they were not polymorphic (except for one).

We make this SNP panel freely available as a resource. As it contains many more untested SNPs, future users will be able to choose the number, density, and position of markers in the linkage map, and considering the local recombination rate around each SNP in order to adjust their own panel to their objectives. This should also be considered for the Massif Central Rivers (in our case Taurion and Seuge Rivers), in which the number of polymorphic loci is lower than in the other basins studied here, probably because of the past demographic histories (colonization, connectivity, stocking, bottlenecks, population sizes and habitat). However, as this subpanel was tested on only 7 populations (190 individuals), we first suggest that increasing the sample size should increase statistical power better than increasing the number of SNPs, especially when Fst is low (< 0.01; [67, 85]).

## Conclusion

The SNP panel presented here appears as a novel tool to study diverse aspects of population genetics in the brown trout. The possibility to genotype many loci in a fast and affordable way will open many perspectives. It opens new insights into the species life history, with many potential applications both for fundamental population genetics, conservation and management questions, but also for more biological questions such as mapping of quantitative trait loci, or investigating links between genetic and environmental divergence. This resource has the potential to offer high flexibility for many possible applications, outperforming previously used markers in many ways: genome coverage and ancestry detection for instance, but also in terms of cost and efficiency to obtain individual genotypes. We hope that it will be useful to the population geneticists’ community working on brown trout and call for future studies across the species’ range.

## Methods

### Development of a genome-wide reference SNP panel

The panel of SNPs markers identified is this study was filtered from the variants described in Leitwein [51] using double-digest RAD sequencing. Restriction enzymes *Eco*RI-HF and *Msp*I were used to digest individual genomic DNA and create the dd-RAD library, which was submitted to size selection in order to retain fragments of 200 to 700 bp using CleanPCR beads. The library was then amplified by PCR and sequenced with Illumina HiSeq2500, producing 125-bp paired-end reads. The initial set contained 75,000 SNPs discovered from 82 wild Mediterranean *S. trutta* from tributaries of the Orb River catchment in southern France, and 102 captive-bred individuals from farms formerly used for stocking in this region (41 and 61 hatchery fish from the ME and AT lineages respectively; see Leitwein [51] for details). These SNPs were anchored to the-high density *S. trutta* linkage map using an intermediate step of physical mapping to the Atlantic salmon reference genome: using their relative positions on the Atlantic salmon reference genome, it was possible to determine the relative mapping positions of a large number of additional RAD loci that were not present on the brown trout linkage map [59].

We applied a series of filters which allowed for selecting a panel of SNP markers that were (i) likely to be highly polymorphic, (ii) mapped on the linkage map, and (iii) present in the two brown trout lineages. To do so, we removed from the initial database all SNPs with a minimum allele frequency (MAF) of 5% or less using *vcftools* [86] based on all individuals (AT + ME). Then, in order to have clean sequences, straightforward to be genotyped, RAD sequences with more than two SNPs, and SNP positions falling in the first or last 30 bp of the RAD sequences were also removed, as well as sequences with undetermined (N) nucleotides. Finally, we kept only RAD sequences for which mapping poisitions on the *S. trutta* linkage map were determined (see above).

### Development of the low-density SNP array

We used this large SNPs resource to develop a low-density SNP array containing 192 SNPs. The goal was to propose a cost-effective tool which holds on two 96-wells genotyping plates for analyzing a large number of individuals. Moreover, SNPs were selected to be informative for population genetics analyses of brown trout populations from at least the AT and ME lineages (sensu Bernatchez [15]) from Western Europe. The array was genotyped using the KASPAR technology® [87] that allows a rapid and cost-effective genotyping service for such a number of markers. We hence applied further filters to fulfill KASPAR genotyping constraints. In particular, RADs with more than one SNP between the primer designing zones (50 bp at the two extremities) were excluded. To ensure a good representativeness of the genome, we selected SNPs evenly spread and spaced by at least 3.5 cM across the 1453 cM estimated length of the *S. trutta* linkage map [59]. This resulted in 245 SNPs (average 1453/245 = 5.9 cM) among which, in order to retain 182 SNPs, we randomly removed 63 SNPs using the *sample()* R function. Then, we added five ancestry informative SNPs developed by the Institut National de Recherche en Agronomie and the University of Savoie (UMR-0042 CARRTEL, France) that were used in previous studies to distinguish individuals from the AT and ME lineages (OMM1164, OMM1105, OMM1154, Str541INRA, Str591INRA; [29, 87–89]).Finally, we added five mitochondrial SNPs previously used to differentiate among the five main brown trout lineages (mitoDA10Proline, mitoDA10ProlineB, mitoCytoB, mitoATPaseIVA, mitoATPaseIVB; [89, 90]; Additional file 1: S1). These numbers of five ancestry informative nuclear or mtDNA markers were chosen to represent approx. 5% of the markers present on the SNP array. This resulted in a low-density array of 192 SNPs markers. Information and sequences are available on Figshare, DOI: 10.6084/m9.figshare.8174708

### Genotyping success of the low-density SNP array

This low-density SNP array was first evaluated for genotyping success using individuals from five independent French river basins: two from the Pyrénées mountains (the Ône and the Aude Rivers), two from the Alps mountains (the Roya and the Doron de Bozel Rivers) and one from the Massif Central mountains (the Seuge River) (Fig. 5). Three of these rivers belong to the Mediterranean lineage (ME; the Aude, the Doron and the Roya Rivers), whereas the two others belong to the Atlantic lineage (AT; the One and the Seuge Rivers) (see Fig. 5).

The sampling sessions were performed in 2016, using a single-pass electrofishing approach from a total of 79 sites (between 8 and 21 sites per river basin, Additional file 1: S5), with an aim of sampling 30 individuals of brown trout per site. In total, we captured 1862 individuals (26 individuals per site in average; see Additional file 1: S5) from which a fin clip was taken (after Eugenol anesthesia), and kept in 70% TE Ethanol for genotyping. All individuals were released alive to their site of capture. Fin samples were sent to the LGC Genomics company for DNA extraction and multilocus genotyping of the 192 SNPs markers using KASPAR® [87]. Genotyping success was measured at the individual level, by the proportion of SNPs which were not genotyped (either because not amplified or because the allele could not be read). Finally, these basins were tested for patterns of isolation-by-distance, by using a mantel test with 1000 permutations on pairwise Fst matrices (calculated with the pairwise.fst *adegenet* function) and riparian distances (measured in meters with STARS ArcGis package).

### Efficiency of the low-density SNPs array

The low-density SNP array was further used for classic population genetic questions in order to compare its efficiency with thirteen microsatellite markers previously used in brown trout population genetic studies (e.g. [91–93]). A total of 190 brown trout individuals were sampled in a small river basin (the Taurion River in the Massif Central Mountains; Fig. 5, “test”) in 2017 using electrofishing. Seven sites were sampled with 21 to 30 individuals per site (Additional file 1: S6). For each individual, a pelvic fin clip was taken for genetic analyses. All individuals were released to their original sampling site. We additionally sampled 30 individuals of domestic Atlantic brown trout from a local hatchery used for stocking purposes (the Soueich trout hatchery), to quantify genetic admixture with wild populations from the Taurion basin. Fin samples were sent to the LGC Genomics Company for DNA extraction and for multilocus genotyping to 192 SNPs markers using the KASPAR® [87]. Note that 30 SNP markers from the initial 192 SNP panel did not amplify (see the Results), and were hence replaced by 30 other SNPs from the 245 SNPs filtered (see methods) to improve the SNP array (see Additional file 1: S2 for details). Additionally, all individuals were genotyped at thirteen microsatellites assembled in PCR multiplexes (see Additional file 1: S7, and Saint-Pé et al., 2018 for details). We tested whether the selected SNPs were likely influenced by selection using the Fst outlier detection method implemented in the *fsthet* R package [95], in which outlier values of *F*_ST_ can be identified in a plot of *F*_ST_ vs. heterozygosity [72].

From both SNP and microsatellite datasets, we removed individuals with more than a third of missing data, and kept only individuals for which we had both SNPs and microsatellites genotypes. We first compared genetic admixture between wild and captive-bred strains using STRUCTURE 2.3.1 [96] with the admixture model and the correlated allele frequency model, without prior population information. Twenty runs assuming two clusters (K = 2, in order to discriminate between wild and captive-bred individuals, see [94]) were performed with a burn-in period of 200,000 and 200,000 subsequent MCMC repetitions. The ten best runs (highest LnP(D) values) were compiled using CLUMPP [97] to obtain final averaged individual Q-values. Individuals were assigned to one of the two clusters with the greatest Q-value, provided that value exceeded 0.7 (as in [94]). Individuals with intermediate Q-values were considered genetically admixed individuals between hatchery and wild strains. Individual assignment Q-values to the cluster containing all Soueich hatchery individuals (i.e. degree of assignment to the “captive-bred” cluster = individual level of “hatchery ancestry”) were compared between SNPs and microsatellites using a Spearman correlation test, as admixed ancestry was not normally distributed.

We then compared population differentiation assessment between markers by calculating pairwise Fst between sites using the *adegenet* R package and *mantel* R function. Finally, we compared the informativeness of both sets of markers for population structure by calculating the informativeness for assignment (*I*_*n*;_ [98]). A higher index indicates a higher informativeness of the set of markers. It was calculated with R as follows: for *i* = 1, 2,..., *K* populations and *m* = 1, 2, ..., *L* loci, with *K* ≥ 2 and *L* ≥ 1. Locus *m* has alleles *j* = 1, 2, ..., *N*_(*m*)_. The average frequency of allele *j* at locus *m* across the *K* populations is defined as *P*_*j*_ = $\sum \limits_{i=1}^K\frac{P_{ij(m)}}{K}$, where *P*_*ij*(*m*)_ is the relative frequency for allele *j* of locus *m* in population *i*. The informativeness is defined as:

$$ {I}_n=\sum \limits_{i=1}^N\left(-{P}_j{logP}_j+\sum \limits_{i=1}^K\frac{P_{ij}}{K}{logP}_{ij}\right) $$

## Additional file

Additional file 1: **S1.** RAD data set of 12204 sequences each containing one or two SNPs, with minimum allele frequency of 5% or higher, no SNPs in the first or last 30 bp of the RAD, no unsequenced nucleotides, and which can be positioned on the *S. trutta* linkage map, **S2.** SNPs used for low density arrays (5 basins and Taurion test). **S3.** Isolation by distance patterns for each river basin, represented by plots of pairwise Fst values against pairwise riparian distance between sites. **S4.** Pairwise Fst values (calculated with adegenet R package) between sites measured with SNPs and with microsatellites. **S5.** Sample sizes and mean (± standard error) body length in mm of fish sampled in each site and each basin for testing the 192 SNPs panel’s genotyping success. **S6.** Map of the Taurion River showing sampling points (black dots). Sample sizes by site, and mean (±SE) body length in mm) from the Taurion River in table. Map was generated by authors on ArcGis and assembled using Inkscape. **S7.** Genotyping microsatellites (DOCX 171 kb)

## Data Availability

SNPs sequences and information are available on Figshare. DOI: 10.6084/m9.figshare.8174708

## Abbreviations

INRA: Institut national de la recherche agronomique
KASPAR: Kompetitive allele-specific PCR amplification of target sequences and endpoint fluorescence genotyping
LD: Linkage disequilibrium
MAF: Minor allele frequency
PCR: polymerase chain reaction
RAD: Restriction site associated DNA
SNP: Single nucleotide polymorphism
