# Supplementary material for: Development of a large SNPs resource and a low-density SNP array for brown trout (Salmo trutta) population genetics
Source: BMC Genomics. 2019 Jul 15;20:582. doi: 10.1186/s12864-019-5958-9 (PMC6631668; doi:10.1186/s12864-019-5958-9)
Supplement: Supplementary file 1 — S1. RAD data set of 12204 sequences each containing one or two SNPs, with minimum allele frequency of 5% or higher, no SNPs in the first or last 30 bp of the RAD, no unsequenced nucleotides, and which can be positioned on the S. trutta linkage map, S2. SNPs used for low density arrays (5 basins and Taurion test). S3. Isolation by distance patterns for each river basin, represented by plots of pairwise Fst values against pairwise riparian distance between sites. S4. Pairwise Fst values (calculated with adegenet R package) between sites measured with SNPs and with microsatellites. S5. Sample sizes and mean (± standard error) body length in mm of fish sampled in each site and each basin for testing the 192 SNPs panel’s genotyping success. S6. Map of the Taurion River showing sampling points (black dots). Sample sizes by site, and mean (±SE) body length in mm) from the Taurion River in table. Map was generated by authors on ArcGis and assembled using Inkscape. S7. Genotyping microsatellites (DOCX 171 kb) [file 12864_2019_5958_MOESM1_ESM.docx]

**A SNP array for brown trout (*Salmo trutta*) population genetics**

Saint-Pé Keoni, Leitwein Maeva, Gagnaire Pierre-Alexandre, Guinand Bruno, Tissot Laurence, Poulet Nicolas, Marselli Geoffrey, Berrebi Patrick, Simon Blanchet

**SUPPLEMENTARY MATERIAL**

*Appendix S1 : RAD data set of 12204 sequences each containing one or two SNPs, with minimum allele frequency of 5% or higher, no SNPs in the first or last 30 bp of the RAD, no unsequenced nucleotides, and which can be positioned on the S. trutta linkage map*

This file contains for each RAD, identification (Locus_ID ), chromosome on Atlantic Salmon CHROMsalar), linkage group on the Brown trout linkage map (LG_Trutta),Position on the brown trout linkage group (Pos_cM_Trutta), positions on the Atlantic salmon chromosome (POSsalar_snp1, POSsalar_snp2), position of the SNP on the Rad tag (POStag_snp1, POStag_snp2), recombination rate ( recombination_rate_cM.Mb), sequence of the tag (TAG), sequencing direction (Orientation), and position of the rad (POS_RAD).

See file «12204RADsFinal.txt»

*Appendix S2: SNPs used for low density arrays (5 basins and Taurion test)*

See file «SNPsLowDensityFinal245.txt»

This file contains for each SNP, identification (SNP_ID), linkage group on the Brown trout linkage map (LG_Trutta), position of the SNP on the Rad tag (POStag_snp), recombination rate (recombination_rate_cM.Mb), sequence of the tag (TAG_sequence).

It also gives success at genotyping information:

- listSuccessTests162 : SNPs which have been genotyped (when TRUE) in the five river basins tested for genotyping success.
- listTaurion182: SNPs which have been genotyped (when TRUE) in the five river basins tested for genotyping success, and added SNPs which have been genotyped in the Taurion basin (after removing the 30 which did not work in the five basins, then the 10 which did not work in the Taurion).

*Appendix S3: Isolation by distance patterns for each river basin, represented by plots of pairwise Fst values against pairwise riparian distance between sites*

*
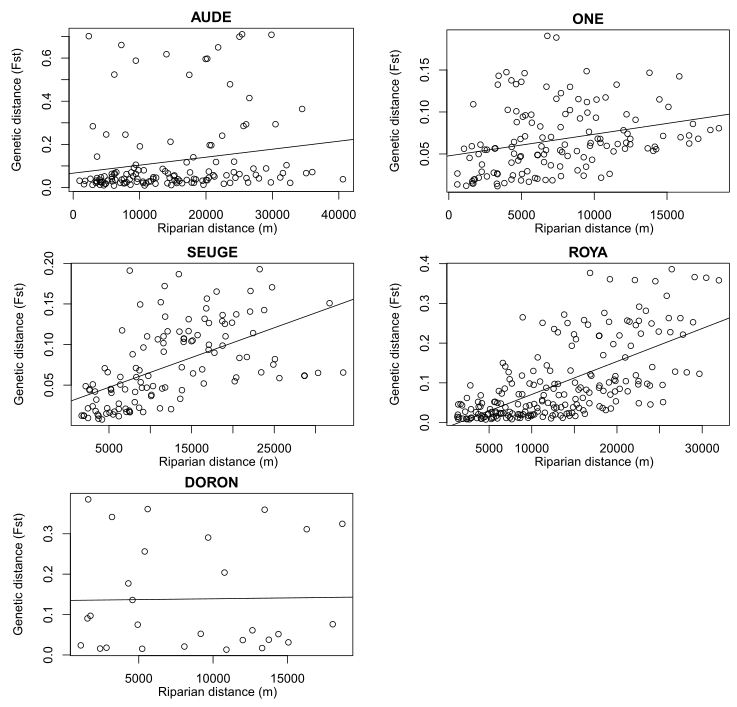
*

*Appendix S4: Pairwise Fst values (calculated with adegenet R package*) *between sites measured with SNPs and with microsatellites*

|  | SNPs | BEA-Rau | PON-Rau | THA-Bar | THA-Tcc | THA-Usi | THA-Vig |
| --- | --- | --- | --- | --- | --- | --- | --- |
| SNPs | PON-Rau | 0.0630 |  |  |  |  |  |
|  | THA-Bar | 0.0579 | 0.0173 |  |  |  |  |
|  | THA-Tcc | 0.0658 | 0.0175 | 0.0175 |  |  |  |
|  | THA-Usi | 0.0695 | 0.0215 | 0.0095 | 0.0193 |  |  |
|  | THA-Vig | 0.0762 | 0.0263 | 0.0126 | 0.0206 | 0.0158 |  |
|  | VIG-Tex | 0.0875 | 0.0337 | 0.0169 | 0.0238 | 0.0120 | 0.0142 |
| Microsatellites | PON-Rau | 0.0389 |  |  |  |  |  |
|  | THA-Bar | 0.0312 | 0.0207 |  |  |  |  |
|  | THA-Tcc | 0.0349 | 0.0115 | 0.0102 |  |  |  |
|  | THA-Usi | 0.0397 | 0.0216 | 0.0127 | 0.0128 |  |  |
|  | THA-Vig | 0.0527 | 0.0329 | 0.0190 | 0.0235 | 0.0121 |  |
|  | VIG-Tex | 0.0489 | 0.0291 | 0.0193 | 0.0222 | 0.0138 | 0.0141 |

*Appendix S5: Sample sizes and mean (± standard error) body length in mm of fish sampled in each site and each basin for testing the 192 SNPs panel’s genotyping success.*

| AUDE | | | | DORON | | | | ONE | | | | ROYA | | | | SEUGE | | | |
| --- | --- | --- | --- | --- | --- | --- | --- | --- | --- | --- | --- | --- | --- | --- | --- | --- | --- | --- | --- |
| Site | N | Mean body length (±SE) | Site | | N | Mean body length (±SE) | Site | | N | Mean body length (±SE) | Site | | N | Mean body length (±SE) | Site | | N | Mean body length (±SE) |  |
| Agu-Sou | 27 | 157(±42) | All-All | | 17 | 126(±55) | Cou-Lar | | 30 | 111(±26) | Ben-Cas | | 8 | 158(±33) | Ber-Ben | | 11 | 114(±28) |  |
| Aig-Pou | 30 | 139(±33) | Boz-Car | | 12 | 173(±56) | Lab-Cas | | 17 | 112(±23) | Bie-Cas | | 30 | 157(±47) | Bui-Pie | | 6 | 96(±27) |  |
| Aig-Sou | 30 | 143(±38) | Boz-Gib | | 15 | 127(±36) | Nga-Jur | | 30 | 152(±40) | Bie-Mai | | 30 | 172(±33) | Cla-Tou | | 9 | 105(±55) |  |
| Art-Lau | 30 | 138(±38) | Boz-Vil | | 18 | 165(±41) | Nga-Mar | | 30 | 135(±18) | Bie-Min | | 30 | 171(±36) | Lav-Riv | | 15 | 138(±51) |  |
| Aud-Car | 30 | 166(±34) | Cha-Chi | | 22 | 133(±40) | Nga-Vga | | 30 | 127(±35) | Cai-Gaf | | 30 | 170(±48) | Lav-Suc | | 30 | 122(±31) |  |
| Aud-Far | 30 | 165(±33) | Chv-Ger | | 23 | 177(±69) | Noo-Ast | | 30 | 135(±26) | Lev-Bri | | 30 | 140(±27) | Pon-Amo | | 30 | 127(±26) |  |
| Aud-Fou | 30 | 135(±25) | Pra-Fra | | 14 | 153(±62) | Noo-Cas | | 30 | 144(±18) | Lev-Ten | | 30 | 153(±30) | Pon-Bom | | 30 | 133(±32) |  |
| Aud-Nen | 30 | 123(±54) | Ros-Mug | | 3 | 137(±72) | Noo-Esp | | 30 | 129(±38) | Mag-Cem | | 30 | 176(±32) | Pon-Cha | | 30 | 148(±29) |  |
| Aud-Puy | 30 | 121(±31) |  | |  |  | Noo-Lac | | 30 | 109(±26) | Ref-Ric | | 30 | 123(±23) | Pon-Tis | | 30 | 129(±18) |  |
| Aud-Ser | 30 | 156(±22) |  | |  |  | Noo-Sav | | 30 | 111(±63) | Roy-Chi | | 24 | 196(±24) | Ser-Sau | | 6 | 143(±28) |  |
| Bai-Pas | 14 | 153(±28) |  | |  |  | Noo-Tre | | 30 | 157(±33) | Roy-Dal | | 30 | 143(±27) | Seu-Cch | | 30 | 110(±21) |  |
| Bru-Mij | 30 | 127(±26) |  | |  |  | Noo-Voo | | 30 | 138(±30) | Roy-Ort | | 30 | 131(±18) | Seu-Cou | | 30 | 134(±27) |  |
| Bru-Uss | 30 | 151(±21) |  | |  |  | Nou-Boo | | 28 | 173(±43) | Roy-Pie | | 21 | 207(±31) | Seu-Cro | | 30 | 165(±33) |  |
| Cam-Sau | 14 | 118(±46) |  | |  |  | Nou-Cir | | 30 | 134(±42) | Roy-Sca | | 30 | 172(±47) | Seu-Fag | | 30 | 126(±32) |  |
| Que-Mas | 29 | 131(±36) |  | |  |  | Nou-May | | 30 | 155(±34) | Roy-Ten | | 30 | 141(±27) | Seu-Rod | | 30 | 157(±38) |  |
| Que-Ria | 30 | 158(±37) |  | |  |  | Nou-Spo | | 30 | 140(±38) | ROY-Fon | | 30 | 199(±65) | Seu-Sau | | 30 | 138(±19) |  |
| Roq-Sau | 9 | 154(±25) |  | |  |  | One-Bag | | 30 | 147(±28) | ROY-Evc | | 30 | 196(±59) |  | |  |  |  |
|  |  |  |  | |  |  |  | |  |  | Roy-Bre | | 30 | 193(±63) |  | |  |  |  |
|  |  |  |  | |  |  |  | |  |  | Roy-Amb | | 30 | 171(±52) |  | |  |  |  |
|  |  |  |  | |  |  |  | |  |  | Roy-Gia | | 30 | 162(±47) |  | |  |  |  |
|  |  |  |  | |  |  |  | |  |  | ROY-Vei | | 30 | 182(±40) |  | |  |  |  |
| Total | 453 | 143(±37) |  | | 124 | 150(±56) |  | | 495 | 136(±38) |  | | 413 | 167(±23) |  | | 377 | 134(±34) |  |

*Appendix S6: Map of the Taurion River showing sampling points (black dots). Sample sizes by site, and mean (±SE) body length in mm) from the Taurion River in table. Map was generated by authors on ArcGis and assembled using Inkscape.*

*
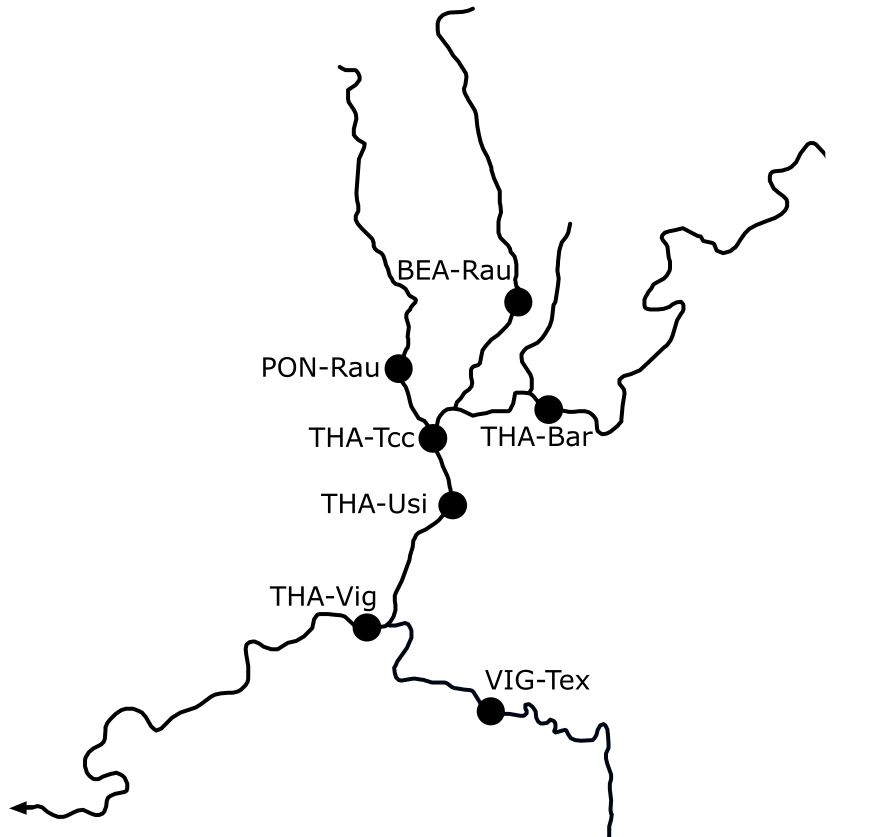
*

| Site | N | Mean (±SE) body length (mm) |
| --- | --- | --- |
| BEA-Rau | 30 | 111(±38) |
| PON-Rau | 21 | 111(±37) |
| THA-Bar | 28 | 221(±108) |
| THA-Tcc | 30 | 144(±46) |
| THA-Usi | 30 | 193(±40) |
| THA-Vig | 26 | 167(±51) |
| VIG-Tex | 25 | 151(±44) |

The Taurion River is a Snow/rain fed stream from the Massif central (France). We sampled between 21 and 30 individuals per site. 4 sites were located on the mainstream (THA sites), the 3 others on tributaries. These sampling sites are represented by black dots on the map.

*Appendix S7: Genotyping microsatellites*

Individual multilocus genotypes were obtained at a total of 13 microsatellite markers (BS131, One9, SSosL311, SsoSL438, T3-13, Sfo1, Ssa064, Ssa103, Ssa417, Ssa-60NVH, Ssa85DU, Ssa-TAP2a and SsoSL417; see Saint-Pé et al. [1] for details on these markers).

The 13 markers were assembled in 3 multiplexes allowing co-amplification of several markers in a single Polymerase Chain Reaction (PCR). Genomic DNA was extracted from the fin clips using a salt-extraction protocol [2]. The loci were amplified using the QIAGEN Multiplex PCR Kit (Qiagen, Valencia, CA, USA). PCR reactions were carried out in a 10 µL final volume containing 5–20 ng of genomic DNA, 5 µL of 2xQIAGEN Multiplex PCR Master Mix, and locus-specific optimized combination of primers. PCR amplifications were performed in a Mastercycler PCR apparatus (Eppendorf, Hauppauge, NY, USA) under the following conditions: 15 min at 95°C followed by 30 cycles of 1 min at 94°C, 1 min at 60°C and 1 min at 72°C and finally followed by a 60 min elongation step at 72°C. Amplified fragments were then separated on an ABI PRISM 3730 automated capillary sequencer. Allelic sizes were finally scored using GENEMAPPER v.4.0 (Applied Biosystems, Foster City, CA, USA). We investigated for anomalies owed to genotyping (e.g. large allele drop; null alleles) using MicrocheckerV 2.2 [3]. As in Saint-Pé et al. [1], we tested for linkage disequilibrium and selection, which none of these 13 loci displayed of.

*References Appendix S7*

1. Saint-Pé K, Blanchet S, Tissot L, Poulet N, Plasseraud O, Loot G, et al. Genetic admixture between captive-bred and wild individuals affects patterns of dispersal in a brown trout (Salmo trutta) population. Conserv Genet. 2018;19:1269–79.

2. Aljanabi S. Universal and rapid salt-extraction of high quality genomic DNA for PCR- based techniques. Nucleic Acids Res. 1997;25:4692–3.

3. Van Oosterhout C, Hutchinson WF, Wills DPM, Shipley P. micro-checker: software for identifying and correcting genotyping errors in microsatellite data. Mol Ecol Notes. 2004;4:535–8.
